# Photoresponsive Polymeric Reversible Nanoparticles via Self-Assembly of Reactive ABA Triblock Copolymers and Their Transformation to Permanent Nanostructures

**DOI:** 10.3390/ma9120980

**Published:** 2016-12-02

**Authors:** Liang Ding, Juan Li, Ruiyu Jiang, Wei Song

**Affiliations:** 1 Department of Polymer and Composite Material, School of Materials Engineering, Yancheng Institute of Technology, Yancheng 224051, China; lijuan@ycit.edu.cn (J.L.); Jiangru@ycit.cn (R.J.); 2 Department of Chemistry, National Taiwan University, Taipei 106, Taiwan

**Keywords:** triblock copolymer, polymeric nanoparticle, photoresponsive material, self-assembly, azobenzene

## Abstract

Azobenzene-functionalized ABA triblock copolymers with controlled molecular weights are prepared first via a sequential ring-opening metathesis polymerization and acyclic diene metathesis polymerization in one-pot, which are readily converted, by a facile esterification, to the modified ABA triblock copolymers. Then, these reactive triblock copolymers can spontaneously self-assemble in a selective solvent to form reproducible and reversible polymeric core-shell nanoparticles. Finally, the stable and permanent shell-crosslinked nanoparticles are obtained by an intramolecular crosslinking reaction in dilute solution under UV light irradiation. These as-prepared polymeric nanoparticles and their precursor incorporating azobenzene chromophores exhibit distinct photoresponsive performance and morphological variation.

## 1. Introduction

The polymeric core-shell nanoparticle is a polymer containing a soluble chain and an insoluble chain in a selective solvent, which has attracted considerable research interest due to the promising applications [1,2]. Many routes to obtain polymeric core-shell nanoparticles with complex structures and functions have been reported. In general terms, the strategy toward a polymeric core-shell nanoparticle can be classified into two categories, that is, the self-assembly of a sequential synthesis of an amphiphilic block copolymer in a selective solvent (the soluble chain could form the shell of a nanoparticle, while the remaining moiety would compose the core of the nanoparticle) [3,4,5,6,7] and the one-pot method to yield nanostructure [8,9,10,11].

For successful synthesis of classical core-shell nanoparticles from the first route, the design and preparation of an amphiphilic block copolymer is critical and of key importance [1]. However, the multi-step synthesis processes limit the usual applicability, though allowing for stimuli-responsive characteristics and further chemical modifications [12]. A different and facile approach was reported by taking advantage of the one-pot polymerization-induced self-assembly [13]. Under certain polymerization conditions, the formation of a diblock copolymer, the aggregation in situ during the chain growth of the second block, and the generation of nanostructure from the aggregation of the resultant copolymer are involved. The chain growth and the self-assembly are interdependent in the polymerization induced self-assembly process. This pathway can make up for the disadvantages of the traditional method, but allow for high designability of monomer and copolymer structures [14,15].

No matter what method is chosen, the synthesis of well-defined block copolymers in principle is essential and requires either living or pseudo-living polymerization chemistry. Increasing attention is now being given to using the diverse polymerizations to prepare the block copolymer precursors first. Recently, we developed a facile one-pot synthesis of unsaturated block polyphosphoester structures by a sequential ring-opening metathesis polymerization (ROMP) and acyclic diene metathesis (ADMET) polymerization. The polymeric nanoparticles were then obtained by spontaneously self-assembly in a selective solvent, which demonstrated multi-functionality and post-functionalization of as-prepared block copolymers [16].

**Scheme 1:**
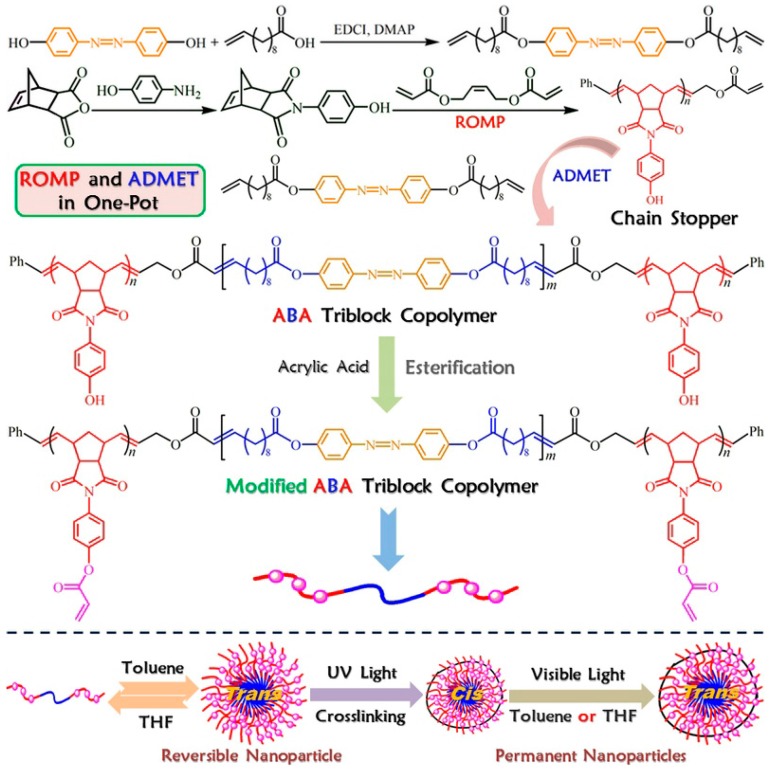
Illustration for preparation of the reactive ABA triblock azobenzene-copolymer and the photoresponsive polymeric core-shell nanoparticle.

The promising results inspired us to further exploit this strategy for the synthesis of a novel azobenzene-functionalized ABA triblock copolymer precursor that could spontaneously self-assemble in a selective solvent to form a polymeric reversible nanoparticle. The hydroxyl groups in ‘A’ blocks were converted to acrylates readily by a simple esterification, and finally fabrication of shell-crosslinked polymeric permanent nanoparticles by intramolecular crosslinking reaction in dilute solution under UV light irradiation (Scheme 1). The morphologies and photoinduced isomerization processes of these polymeric core-shell nanoparticles displayed unique variations when azo chromophores in the core underwent *trans–cis–trans* isomerization cycles.

## 2. Results and Discussion

### 2.1. Preparation of the Reactive ABA Triblock Copolymer Precursor via One-Pot Sequential ROMP and ADMET Polymerization

**Figure 1:**
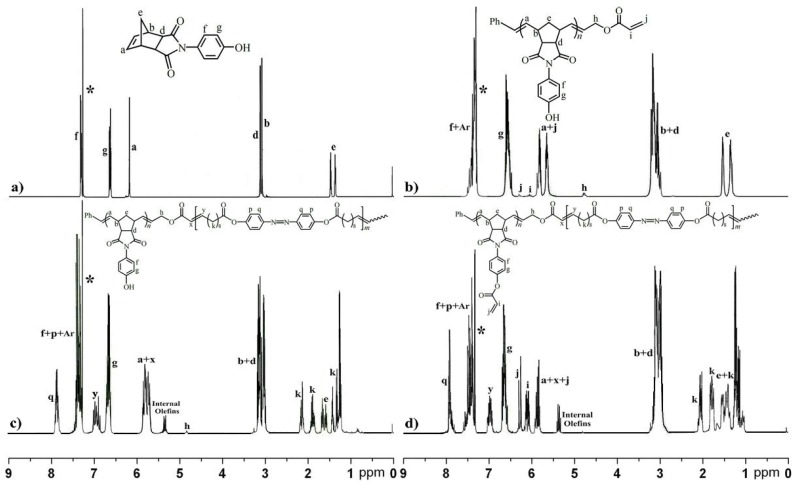
^1^H NMR spectra for (**a**) ROMP monomer; (**b**) ROMP homopolymer; (**c**) ABA triblock copolymer; and (**d**) the reactive triblock copolymer precursor (* CDCl_3_).

**Figure 2:**
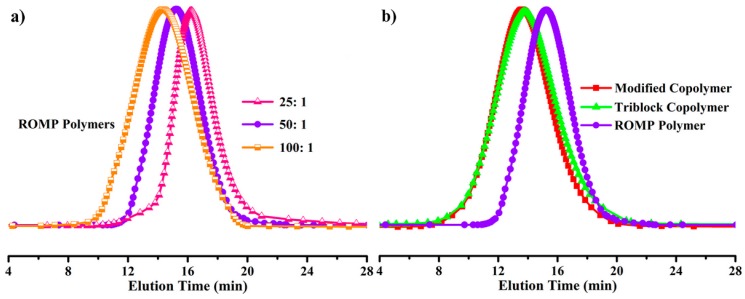
Representative GPC traces of (**a**) ROMP homopolymers with different ratio of monomer and catalyst and (**b**) ROMP homopolymer, ABA triblock copolymer, and the reactive ABA triblock copolymer precursor.

As shown in Scheme 1, the reactive ABA triblock copolymer precursor was successfully prepared by a three-step technique. As the first step, a novel versatile ROMP monomer bearing a cyclic norbornenyl group and a hydroxyl group was synthesized according to the previous reports [17]. ^1^H NMR spectroscopy (Figure 1a) and elemental analysis affirmed the structure and purity of the ROMP monomer. Then, we investigated ROMP in the presence of a functional terminating agent (TA). One of the advantages in using ROMP with functionalized symmetrical TA for the preparation of monotelechelic polymer is the ability to directly incorporate the desired functional group onto the polymer by reaction with the living chain end without need to perform the complicated post-polymerization transformations. Because the backbone olefins of substituted cyclic monomer (poly(oxa)norbornenes) are too sterically hindered to undergo metathesis, the TA adds only to the *ω*-end of the polymer [18]. The polymerization conditions and results are listed in Table 1. The unimodal GPC traces in Figure 2a are observed for these ROMP polymers with a relatively reasonable molecular weight distribution, and gradually shifted to a much higher molecular weight region with the increase in the monomer/catalyst molar ratio (25:1~100:1). Moreover, the structure of the ROMP polymer was confirmed by NMR spectroscopy. In ^1^H NMR spectrum, the alkene signals shifted from 6.17 ppm (H_a_) in Figure 1a to approximately 5.91–5.49 ppm (H_a_) in Figure 1b after ROMP, indicating the polymerization had occurred. Unfortunately, the capping efficiency was not evaluated due to the characteristic phenyl protons on polymer chain end overlapped. However, we can calculate the number-average molecular weight (*M*_n_) of the ROMP homopolymer by comparing the peak integration areas of phenyl protons on each monomer unit at 6.72–6.39 ppm (*S*_g_, 2H) with that of methylene protons adjacent to acrylate group at the end of the polymer backbone at 4.81–4.74 ppm (*S*_h_, 2H). *M*_n_ could be determined through a formula: *M*_n,NMR_ = (*S*_g_/*S*_h_) × *M*_(monomer)_ + *M*_(TA)_/2 + *M*_(Ar-CH)_, which are in good agreement with the values obtained from GPC traces (Table 1).

Meier et al. have reported that mono- or multi-functional acrylate (macro)molecule could be considered as a selective and irreversible chain stopper in ADMET polymerization [19,20,21]. That is to say, as-prepared ROMP polymer containing terminal acrylate functional group could act as a macromolecular. Therefore, diene monomer and **C2** were added to the aforementioned ROMP system to make the polymerization transform into ADMET polymerization, constructing ABA triblock copolymer in a one-pot process. The analysis of the resulting product by ^1^H NMR (Figure 1c) detected the new internal unsaturated olefin signals at 5.47–5.35 ppm, indicating ADMET polymerization occurred indeed. Importantly, the new formed α,β-unsaturated ester functions at 7.01–6.96 (H_y_) and 5.92–5.71 ppm (H_x_) was also observed, meanwhile, the terminal olefin signals at 4.99 ppm disappeared, illustrating the ROMP polymer was linked to two ends of the ADMET polymer. Using a similar calculation method, *M*_n,NMR_ of ‘B’ block was determined via a formula: *M*_n,NMR_ = (*S*_(internal olefin)_/*S*_h_) × *M*_(monomer)_, which was further used to calculate *M*_n,NMR_ of ABA triblock copolymer (*M*_n,NMR_ = 2 × *M*_(A block)_ + *M*_(B block)_ − 2 × *M*_(ethylene)_). GPC trace in Figure 2b displayed an evidently increased molecular weight when compared with the ROMP homopolymer, and the value was close to *M*_n,NMR_. All these results clearly demonstrate that this one-pot strategy is feasible to prepare ABA triblock copolymer via ADMET polymerization by growing a second (third) block ‘step-by-step’ from a selective macromolecular chain stopper obtained from ROMP. There are lots of pendant hydroxyl groups in ‘A’ blocks, which could be converted to acrylate by a simple esterification, finally forming the reactive ABA triblock copolymer with acrylate pendants. The precise structure can be determined by ^1^H NMR spectrum (Figure 1d) according to the resonance signals of acrylate protons (H_i_, H_j_) at 6.39–5.97 ppm. Using a similar method, *M*_n,NMR_ of this copolymer was calculated and shown in Table 1. Noticeably, the molecular weight and molecular weight distribution of the reactive triblock copolymer precursor are almost the same as the ABA triblock copolymer, confirming the successful transformation of hydroxyl into acrylate. Although complete conversion may not be attained, it has been enough to utilize for self-crosslinking.

### 2.2. Determination of the Polymeric Nanoparticles Formed in a Selective Solvent

The micellization of the reactive triblock copolymer with a constant shell and core lengths in the selective solvent of toluene was performed successfully. It is worthwhile to note that only two ROMP homopolymers (‘A’ blocks) are totally soluble in toluene, while the other ADMET homopolymer (‘B’ block) is insoluble in toluene. Consequently, the solubility discrepancy between these two blocks in copolymers provides an opportunity of self-aggregation to form the nanoparticles in toluene [11]. The size and morphology of aggregated nanoparticles were investigated by means of DLS, AFM, and TEM techniques.

**Figure 3:**
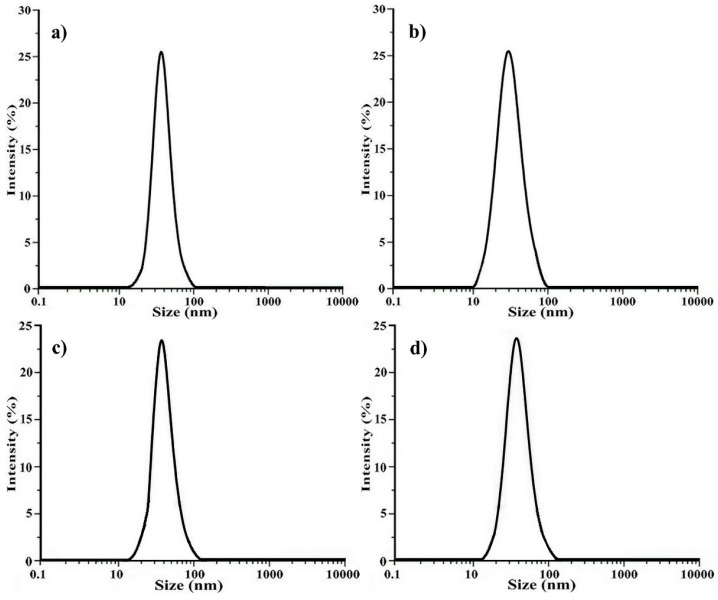
Sizes and distributions of nanoparticles determined by DLS: (**a**) the polymeric core-shell nanoparticles were dried and redissolved in toluene; (**b**) the original toluene solution of the shell-crosslinked nanoparticles; (**c**) after drying and redissolving in toluene; and (**d**) in THF.

**Figure 4:**
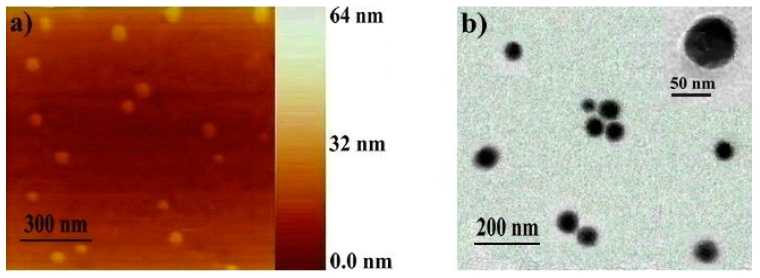
Polymeric nanoparticles formed by self-assembly of the reactive triblock copolymer precursor in toluene (**a**) AFM image and (**b**) TEM image.

DLS data demonstrated that the triblock copolymer was nicely formed aggregates in toluene, and the average hydrodynamic diameter was measured to be 92 nm. Meanwhile, the nanoparticles showed homogenous distributions with the low polydispersity index (PDI) value of 0.165 (Table 2 and Figure 3a). Additionally, there had been basically the same size and distribution after storing the sample for one week or even longer, or drying and redissolving it in toluene, indicating the nanoparticles were actually stable and reproducible in toluene. However, the aggregates would disappear in other solvents such as THF that both ‘A’ and ‘B’ blocks could dissolve. The morphology of polymeric nanoparticles in toluene was preliminarily examined by AFM, which exhibited many separate and randomly deposited particles with a diameter of around 85 nm, and also there are a few large particles the size of several hundred nanometers (Figure 4a), perhaps caused by global micelles overlapping during the solvent evaporation process. The morphology of polymeric nanoparticle from copolymer was further confirmed by TEM, and the representative photogram was shown in Figure 4b. It can be seen that most of the well-dispersed spherical nanoparticles have an average diameter of 82 nm in dried state, while some aggregated structures exist, which is coincident with the AFM result, but smaller than the size detected by DLS due to the solvated effect [22].

### 2.3. Crosslinking of the Shell of Polymeric Core-Shell Nanoparticles by UV Light Irradiation

It is well known that acrylate group can easily react through UV light irradiation or thermal conditions [20,23,24,25]. Therefore, polymers bearing acrylate groups are widely used in the preparation of crosslinked or surface modification materials without adding any other small molecular crosslinking agents. However, when the reaction is performed under an ultradilute solution, intramolecular crosslinked polymer would be obtained. Bearing acrylate pendants in ‘A’ blocks, the shell of the nanoparticle could be crosslinked under UV light irradiation by intramolecular reaction at the dilute concentration. After UV light irradiation of the solution in toluene, the size and distribution of shell-crosslinked nanoparticle (original solution sample) changed distinctly.

**Figure 5:**
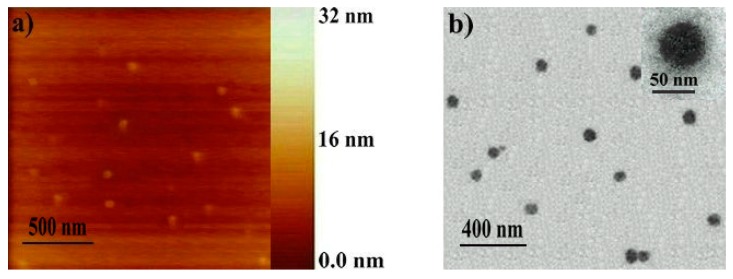
The original solution sample of the shell-crosslinked nanoparticles in toluene (**a**) AFM image and (**b**) TEM image.

**Figure 6:**
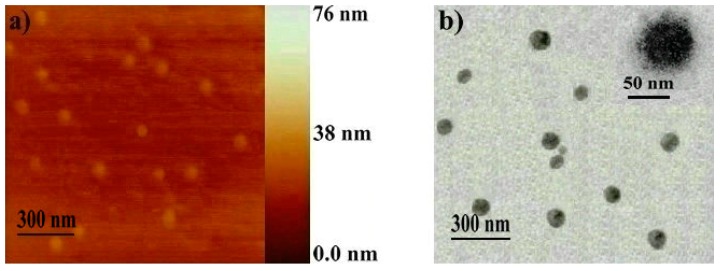
Drying and redissolving of the shell-crosslinked nanoparticles in THF (**a**) AFM image and (**b**) TEM image.

DLS detected hydrodynamic diameter of the shell-crosslinked polymeric nanoparticles was 64 nm, and PDI was 0.182 in toluene (Figure 3b). The UV light irradiated sample was then dried and redissolved in THF which is a nonselective but a good solvent for the triblock copolymer, and this THF solution was used to further investigate the photo-crosslinking effect. As expected, the diameter of 110 nm and a PDI of 0.191 with the monomodal size-distribution were afforded by DLS measurement (Figure 3d), which were slightly larger than those before crosslinking. This presumably is attributed to the swelling of the particles in the nonselective solvent. These results indicated that the photo-crosslinking locked the shell of preformed nanoparticles, causing a permanently fixed structure of nanoparticle. The morphology of the shell-crosslinked nanoparticle was also investigated by means of AFM and TEM. As shown in Figure 5a, we can see many deposited particles on the mica surface as a spherical morphology with an average diameter of 56 nm. TEM image also showed some separated spherical nanoparticles with an average diameter of 50 nm in a comparatively uniform size (Figure 5b). After drying and redissolving in THF, AFM and TEM images (Figure 6) supported the formation of the shell-crosslinked permanent nanoparticles which have an increased size of about 93 and 98 nm, respectively.

The obvious change in size was actually correlated with azobenzene groups in the core of nanoparticle. It is well-known that polymers containing azobenzene-type chromophores have unique reversible photoresponsive *trans*–*cis–trans* isomerization under UV and visible lights irradiation [26]. Generally, the *trans* form of the azobenzene molecule is thermodynamically stable than the *cis* [26,27]. Herein, the initial state of azobenzene groups in polymeric nanoparticle was the *trans* isomer, however, the *cis* isomer was obtained during the process of photo-crosslinking under UV light irradiation. In other words, azobenzene groups in shell-crosslinked polymeric nanoparticles were the *cis* isomers, leading to decrease in size. When storing a short time in visible light (or the process of drying and redissolving), the *cis* form would change into the *trans* form, resulting in increased size also [28]. Of course, if the sample in THF was irradiated by UV light once again for a period of time, the diameter of nanoparticles would also reduce to around 65 nm due to the *trans* to *cis* photoisomerization process.

### 2.4. Photoresponsive Property of the Reactive ABA Triblock Copolymer Precursor and the Polymeric Shell-Crosslinked Nanoparticles in Different Selective Solvents

**Figure 7:**
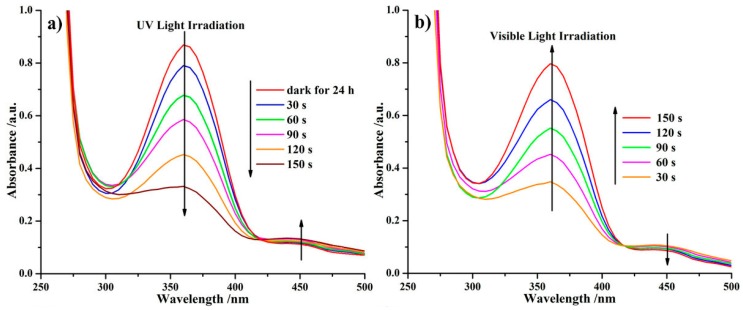
Typical UV–vis spectral changes of copolymer precursor in THF solution under (**a**) UV light irradiation (365 nm) and (**b**) visible light.

**Figure 8:**
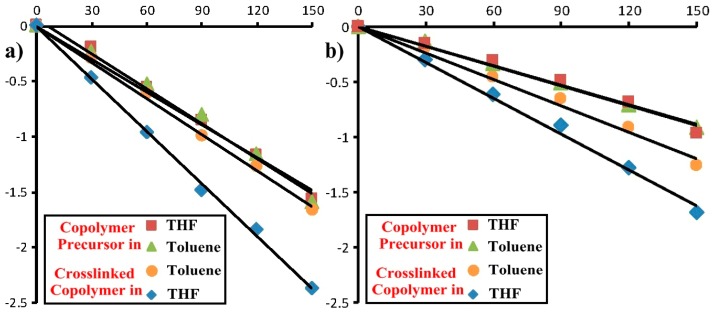
The first-order kinetics of copolymer precursor and crosslinked copolymer in different solutions: (**a**) *trans–cis* photoisomerization and (**b**) *cis–trans* back-isomerization.

Since the prepared triblock copolymer embedded with azobenzene moieties can undergo reversible *trans*–*cis*–*trans* photoisomerization, the photoresponsive properties of these copolymers were investigated in dilute THF solution through UV–Vis absorption spectra at selected time intervals with varying conditions of UV or visible light irradiation [29]. As illustrated by the UV–Vis spectra in Figure 7, the solution of ABA triblock copolymer in THF underwent *trans* to *cis* photoisomerization (Figure 7a) and *cis* to *trans* back-isomerization (Figure 7b). When it dissolved in toluene, the triblock copolymer self-assembled to nanoparticles bearing azobenzene groups in core. Once the processes used in Figure 8a,b were repeated, there were no obvious characteristic absorption peak changes compared with those in THF, but the UV isomerization efficiency at the same time intervals for photoisomerization was evidently different. However, after crosslinking under UV light irradiation, for the shell-crosslinked nanoparticles, UV–Vis spectra of initial reaction solution in toluene displayed that the sample reached the *cis* photostationary state, which was just to make an explaination for obtaining small size nanoparticles. Upon visible light irradiation, the solution would undergo *cis* to *trans* photoisomerization. If the original reaction solution was dried and redissolved in THF, UV–vis spectra showed the *trans* photostationary state because the *trans* form is thermodynamically more stable than the *cis* form, which was also according with the large size of nanoparticles. However, isomerization efficiency decreased.

In order to explain these differences of isomerization efficiency, the first-order kinetics curves of *trans*–*cis* and *cis*–*trans* recovery of these copolymers in toluene and THF are also plotted in Figure 8, and the corresponding rate constants (*k*_l_ and *k*_r_) were calculated and summarized in Table 2. It was worth noting that the polymeric shell-crosslinked nanoparticle displayed similar rate constants in THF and toluene, but slower photoresponse compared with the starting triblock copolymer precursor, while the copolymer precursor in toluene showed slower photoresponse than that in THF because of its more compact chain entanglement. In brief, for the polymeric nanoparticles, azobenzene groups in the core are in so confined a space that there is not enough space to fulfil the isomerization processes of these chromophores, leading to great hindrances in the process of *trans*–*cis*–*trans* photoisomerization.

## 3. Materials and Methods

### 3.1. Materials

5-Norbornene-endo,endo-2,3-dicarboxylicanhydride (98%), 4,4′-dihydroxyazobenzene (>98%), dichloro[1,3-bis(2,4,6-trimethylphenyl)-2-imidazolidinylidene](benzylidene)bis(3-bromopyridine)ruthenium(II) (Grubbs third generation catalyst, **C1**) (95%), 4-aminophenol (96%), [1,3-bis(2,4,6-trimethylphenyl)-2-imidazolidinylidene]dichloro(o-isopropoxyphenylmethylene)-ruthenium (Hoveyda–Grubbs second generation catalyst, **C2**) (98%), acrylic acid (99%), 1-[3-(Dimethylamino)propyl]-3-ethylcarbodiimide hydrochloride (EDCI·HCl, >99%), oxalyl chloride [(COCl)_2_, (98%)], 4-dimethylaminopyridine (DMAP) (98%), and benzoin (99%) were purchased from Energy Chemical (Shanghai, China) and used as received without purification. Solvents were distilled over drying agents under nitrogen prior to use. Triethylamine (Et_3_N) was freshly distilled and dried by molecular sieves. 1,4-Diacrylate-*cis*-2-butene was synthesized as reported previously in our group.

### 3.2. Characterization

^1^H NMR spectra were recorded on a Varian 400 Unity Plus spectrometer (Billerica, MA, USA), a Bruker AVIII-500 MHz, or a Bruker AVIII-800 MHz FT-NMR spectrometer (Billerica, MA, USA) at ambient temperature. Relative molecular weights and molecular weight distributions were measured by gel permeation chromatography (GPC) equipped with a Waters 1500 Isocratic HPLC pump, a Waters 2414 refractive index detector, and a set of Waters Styragel columns (7.8 × 300 mm^2^, 5 μm bead size; 10^3^, 10^4^, and 10^5^ Å pore size) (Milford, MA, USA). GPC measurements were carried out at 35 °C using THF as the eluent with a flow rate of 1.0 mL/min. The system was calibrated with polystyrene standards. The UV–Vis absorption spectra were measured on an Agilent Cary 60 spectrometer (Palo Alto, CA, USA). UV irradiation was carried out with an 8 W × 4 UV lamp with the wavelength at 365 nm. Irradiation by visible light was performed using a 23 W Philips day light bulb (>400 nm). The elemental analysis (EA) was conducted with an Elementar vario EL. The hydrodynamic diameter was determined by the dynamic light scattering (DLS) analysis using a Malvern Zetasizer Nano-ZS light scattering apparatus (Malvern Instruments, Worcestershire, UK) with a He-Ne laser (633 nm, 4 mW). Atom force microscopy (AFM) observations were performed on an SPM AJ-III atomic force microscope at a measurement rate of 1.0005 Hz in the tapping mode, and the AFM images were obtained at room temperature in air. The samples for transmission electron microscopy (TEM) were prepared by depositing a drop of the solution on a carbon-coated Cu grid, and TEM images were recorded on the FEI Tecnai G2 F30 microscopes (Hillsboro, OR, USA) operating at an acceleration voltage range of 50–300 kV.

### 3.3. Sample Preparation for AFM and TEM

The morphology of nanoparticles was first observed by AFM. An ultrathin film of micelle was prepared by pouring the original copolymer solution with a concentration of 0.03 mg/mL onto freshly cleaved mica surface, which was then air-dried at room temperature. The sample was examined at least twice under the same conditions, and the images were found to be reproducible.

A sample for TEM measurement was prepared by pouring the original copolymer solution with a concentration of 0.03 mg/mL onto a carbon-coated copper grid, followed by air-drying at room temperature. For shell-crosslinked nanoparticles, the sample was detected under UV or visible light irradiation.

### 3.4. Synthesis of 4,4′-bis(Undecylenyloxycarbonyl) Azobenzene (Monomer for ADMET Polymerization)

To a Schlenk flask, 4,4′-dihydroxyazobenzene (2.14 g, 10 mmol), 10-undecenoic acid (4.05 g, 22 mmol), and DMAP (0.3 g, 2.4 mmol) were dissolved in dried CH_2_Cl_2_ (35 mL) and THF (5 mL), and the mixture was stirred at 0 °C for 15 min. EDCI (4.59 g, 24 mmol) was then added to the former solution, and stirred for 72 h under nitrogen flow after the solution was warmed to room temperature. The resulting solution was washed three times with deionized water (3 × 80 mL), and the organic layer was dried over anhydrous Na_2_SO_4_. The solvent was then evaporated, and the crude product further purified by recrystallization from CH_3_OH/THF (4/1) to give a red crystal (4.11 g, 75.2% yield).

### 3.5. Synthesis of N-4-Phenol-Norbornene-Dicarboximide (Monomer for ROMP)

A solution of 5-norbornene-endo,endo-2,3-dicarboxylicanhydride (3.28 g, 20 mmol) and 4-aminophenol (2.18 g, 20 mmol) in toluene (40 mL) was added to a dried, 100 mL round-bottom flask fitted with a Dean-Stark trap and placed in an oil bath preset to 140 °C for 24 h while stirring. The reaction mixture was concentrated under reduced pressure, diluted with CH_2_Cl_2_ (100 mL), and washed with three times with deionized water (3 × 50 mL). The organic layer was dried over anhydrous Na_2_SO_4_, filtered, and evaporated. The crude product was purified by recrystallization from CH_3_OH/CHCl_3_ (5/1) to give a white crystalline solid (4.11 g, 80.6% yield).

### 3.6. One-Pot Synthesis of Acrylate-Terminated Monotelechelic Polymer (Macromolecular Chain Stopper) and ABA Triblock Copolymer

In a nitrogen-filled Schlenk tube, a solution of Grubbs third generation catalyst (1 equiv.) in THF was degassed with three freeze–vacuum–thaw cycles and then added to a degassed (with the same procedure as above) solution of above monomer (50 equiv.) in THF, to give a monomer concentration of 0.5 mol/L. After stirring at 50 °C for 12 h under nitrogen flow, terminating agent, 1,4-diacrylate-*cis*-2-butene (10 equiv.) was added as a solution in THF. After the reaction being conducted under nitrogen atmosphere at 50 °C for 24 h, a first aliquot (~1.0 mL) of the solution was taken out from the reaction mixture by syringe and the polymerization was stopped, and then the content was poured into an excess methanol. The precipitate was isolated and dried under vacuum for 24 h to provide the monotelechelic ROMP polymer.

After that, a solution of azobenzene-functionalized symmetric *α*,*ω*-diene monomer (*α*,*ω*-diene monomer and monotelechelic ROMP polymer in 10:1 molar ratio) and Hoveyda–Grubbs second generation catalyst (0.5 mol % to *α*,*ω*-diene monomer) in THF degassed with the same procedure was added to above ROMP system to make the polymerization transform into ADMET polymerization, and the reaction mixture was stirred at 70 °C for another 24 h under a slow purge of nitrogen to drive off the ethylene condensate. The polymerization was finally quenched by adding ethyl vinyl ether with stirring for 30 min. The content was precipitated in excess methanol, and the precipitate was isolated by filtration and dried under vacuum for 24 h to yield the triblock copolymer (yield: 92%).

### 3.7. Transformation of Hydroxyl Group of ABA Triblock Copolymer via Esterification to Reactive Triblock Copolymer Precursor

Under a nitrogen atmosphere, (COCl)_2_ (180 mmol) was added by syringe to acrylic acid (18 mmol) at room temperature with rapid stirring. After 6 h, the excess (COCl)_2_ was removed in vacuo to yield acryloyl chloride, which was dissolved in 10 mL of THF, and then added by syringe to the solution of as-prepared triblock copolymer (0.1 mmol) in 2 mL of THF and dry triethylamine (0.5 mmol) at 0 °C. The reaction mixture was then allowed to warm to room temperature and stirred overnight. The product was then precipitated quantitatively twice from water and methanol and dried for 24 h in a vacuum oven to afford a reactive triblock copolymer precursor (yield: 95%).

### 3.8. UV-Crosslinking of Polymeric Core-Shell Nanoparticle Solution from Reactive Triblock Copolymer Precursor

The intramolecular cross-link of the reactive ABA triblock copolymer precursor was typically performed as follows: the reactive triblock copolymer precursor was dissolved in toluene in a dry round-bottom flask under a nitrogen atmosphere to form the polymeric nanoparticle solution. The reaction mixture was degassed under vacuum, and then a catalytic amount of benzoin (10 wt %) was added. Subsequently, the flask was placed in a UV curing box (400 W Hg lamp, 365 nm) for 15 min under stirring. The solution was concentrated and precipitated into an excess of methanol; the product was isolated by filtration and dried under vacuum for 24 h to yield the shell-crosslinked nanoparticle copolymer as a solid (yield, >95%).

## 4. Conclusions

The well-defined ABA triblock copolymer was successfully first prepared in a controlled way on the basis of tandem catalysis using Grubbs ruthenium-based catalysts to sequentially mediate ROMP and ADMET polymerization in one-pot. The reactive triblock copolymer precursor was then readily obtained by an esterification, which could self-assemble into the polymeric core-shell nanoparticles only in the selective solvent of toluene. The shell of nanoparticles bearing multi-acrylate groups could be further intramolecularly crosslinked in the dilute condition under UV light irradiation to finally produce the shell-crosslinked nanoparticles. The sizes and morphologies of these aggregates were investigated through DLS, AFM, and TEM measurements, which displayed unique transformations when azobenzene chromophores in the core of these nanoparticles underwent *trans*–*cis*–*trans* isomerization. The photoisomerization processes of these copolymers were monitored using UV–Vis spectroscopy upon irradiation with UV or visible light, which presented a different isomerization efficiency. The first-order kinetic parameters for these two isomerization processes were calculated, which were used to explain the differences in the isomerization efficiency and the sizes of nanoparticles. Further research is to optimize the structure of ABA triblock copolymer precursor and construct the polymeric nanoparticles composed of the core of ‘A’ blocks and the shell of ‘B’ block in a selective solvent, and finally obtaining the polymeric core-crosslinked nanoparticles.

## Figures and Tables

**Table 1:** Characteristics of homopolymer by ROMP, ABA triblock copolymer via ADMET polymerization, and triblock copolymer precursor through esterification.

Polymer	Yield ^1^ (%)	[M]/[C] ([M]/[CS]) ^2^	*M*_n,GPC_^3^	*M*_w_/*M*_n_^3^	*M*_n,NMR_	*DP*_n,ROMP_^6^	*DP*_n,ADMET_^7^
Homopolymer	92	25:1	8500	1.68	7100 ^4^	27.2	–
	93	50:1	13,700	1.59	12,800 ^4^	49.3	
	89	100:1	26,300	1.65	24,200 ^4^	94.2	
Copolymer	86	10:1	34,900	1.76	32,300 ^5^	49.3	11.8
Precursor	96	–	36,200	1.75	–	49.3	11.8

^1^ Obtained gravimetrically from the dried polymer; ^2^ [M]/[C] was the feed molar ratio between ROMP monomer and Grubbs catalyst; [M]/[CS] was the feed molar ratio between ADMET monomer and chain stopper (ROMP homopolymer); ^3^ Number-average molecular weight (*M*_n_) and molecular weight distribution (*M*_w_/*M*_n_) were determined by GPC in THF relative to monodispersed polystyrene standards; ^4^ Obtained by ^1^H NMR spectroscopy: *M*_n,NMR_ = (*S*_g_/*S*_h_) × *M*_(monomer)_ + *M*_(TA)_/2 + *M*_(Ar-CH)_, where *M*_(monomer)_ = 255, *M*_(TA)_ = 196, and *M*_(Ar-CH)_ = 90; ^5^ Obtained by ^1^H NMR spectroscopy: *M*_n,NMR_ = (*S*_(internal olefin)_/*S*_h_) × *M*_(monomer)_ + 2 × *M*_(homopolymer)_, where *M*_(monomer)_ = 544; ^6^ Calculated by ^1^H NMR spectroscopy: *DP*_n_ = *S*_g_/*S*_h_; ^7^ Calculated by ^1^H NMR spectroscopy: *DP*_n_ = *S*_(internal olefin)_/*S*_h_.

**Table 2:** Sizes and distributions of polymeric nanoparticles, kinetic parameters for the *trans–cis–trans* photoisomerization in different selective solvents.

Polymer	Solvent	*k*_i_^6^	*k*_r_^7^	DLS ^8^		AFM (nm)	TEM (nm)
		(S-1/10-2)		*D*_h_ (nm)	PDI		
Copolymer Precursor	THF ^1^	1.59	1.06	–		–	
	Toluene ^2^	1.08	0.74	92	0.165	85	82
Crosslinked Copolymer	Toluene ^3^	–		64	0.182	56	50
	Toluene ^4^	0.99	0.58	108	0.189	–	
	THF ^5^	1.02	0.60	110	0.191	93	98

^1^ The original THF reaction solution; ^2^ After drying and redissolving in toluene; ^3^ The original toluene solution; ^4^ After drying and re-dissolving in toluene; ^5^ After drying and re-dissolving in THF; ^6^ The rate constant of *trans*–to–*cis* photoisomerization; ^7^ The rate constant of *cis*–to–*trans* photoisomerization; ^8^ The average diameter (*D*_h_) and the polydispersity (PDI) of nanoparticles in different solvents were determined by DLS technique.
